# Digital Behavior Change Interventions for the Prevention and Management of Type 2 Diabetes: Systematic Market Analysis

**DOI:** 10.2196/33348

**Published:** 2022-01-07

**Authors:** Roman Keller, Sven Hartmann, Gisbert Wilhelm Teepe, Kim-Morgaine Lohse, Aishah Alattas, Lorainne Tudor Car, Falk Müller-Riemenschneider, Florian von Wangenheim, Jacqueline Louise Mair, Tobias Kowatsch

**Affiliations:** 1 Future Health Technologies Programme Campus for Research Excellence and Technological Enterprise Singapore-ETH Centre Singapore Singapore; 2 Saw Swee Hock School of Public Health National University of Singapore Singapore Singapore; 3 Centre for Digital Health Interventions Institute of Technology Management University of St Gallen St Gallen Switzerland; 4 Centre for Digital Health Interventions Department of Management, Technology, and Economics ETH Zurich Zurich Switzerland; 5 Lee Kong Chian School of Medicine Nanyang Technological University Singapore Singapore; 6 Department of Primary Care and Public Health School of Public Health Imperial College London London United Kingdom; 7 Yong Loo Lin School of Medicine National University of Singapore Singapore Singapore

**Keywords:** digital health companies, health care, type 2 diabetes, prevention, management, conversational agent, digital behavior change intervention, investment, just-in-time adaptive intervention, digital health, diabetes, agent, behavior

## Abstract

**Background:**

Advancements in technology offer new opportunities for the prevention and management of type 2 diabetes. Venture capital companies have been investing in digital diabetes companies that offer digital behavior change interventions (DBCIs). However, little is known about the scientific evidence underpinning such interventions or the degree to which these interventions leverage novel technology-driven automated developments such as conversational agents (CAs) or just-in-time adaptive intervention (JITAI) approaches.

**Objective:**

Our objectives were to identify the top-funded companies offering DBCIs for type 2 diabetes management and prevention, review the level of scientific evidence underpinning the DBCIs, identify which DBCIs are recognized as evidence-based programs by quality assurance authorities, and examine the degree to which these DBCIs include novel automated approaches such as CAs and JITAI mechanisms.

**Methods:**

A systematic search was conducted using 2 venture capital databases (Crunchbase Pro and Pitchbook) to identify the top-funded companies offering interventions for type 2 diabetes prevention and management. Scientific publications relating to the identified DBCIs were identified via PubMed, Google Scholar, and the DBCIs’ websites, and data regarding intervention effectiveness were extracted. The Diabetes Prevention Recognition Program (DPRP) of the Center for Disease Control and Prevention in the United States was used to identify the recognition status. The DBCIs’ publications, websites, and mobile apps were reviewed with regard to the intervention characteristics.

**Results:**

The 16 top-funded companies offering DBCIs for type 2 diabetes received a total funding of US $2.4 billion as of June 15, 2021. Only 4 out of the 50 identified publications associated with these DBCIs were fully powered randomized controlled trials (RCTs). Further, 1 of those 4 RCTs showed a significant difference in glycated hemoglobin A_1c_ (HbA_1c_) outcomes between the intervention and control groups. However, all the studies reported HbA_1c_ improvements ranging from 0.2% to 1.9% over the course of 12 months. In addition, 6 interventions were fully recognized by the DPRP to deliver evidence-based programs, and 2 interventions had a pending recognition status. Health professionals were included in the majority of DBCIs (13/16, 81%,), whereas only 10% (1/10) of accessible apps involved a CA as part of the intervention delivery. Self-reports represented most of the data sources (74/119, 62%) that could be used to tailor JITAIs.

**Conclusions:**

Our findings suggest that the level of funding received by companies offering DBCIs for type 2 diabetes prevention and management does not coincide with the level of evidence on the intervention effectiveness. There is considerable variation in the level of evidence underpinning the different DBCIs and an overall need for more rigorous effectiveness trials and transparent reporting by quality assurance authorities. Currently, very few DBCIs use automated approaches such as CAs and JITAIs, limiting the scalability and reach of these solutions.

## Introduction

In 2019, approximately 463 million adults were estimated to be living with diabetes [1]. This estimate is expected to rise to more than 700 million by 2045 [1]. More than 90% of this burden is caused by type 2 diabetes. [1,2]. Over 1 million deaths worldwide were attributed to this condition in 2017 alone, making it the ninth leading cause of mortality [3]. Diabetes is also a leading source of global health expenditure with an estimated annual cost of US $760 billion in high- and low-income countries, including the United States (US $259 billion), China (US $109 billion), and Brazil (US $52 billion) [1].

Guidelines for the prevention and management of type 2 diabetes include specific recommendations for lifestyle behavior changes such as diet, exercise, smoking cessation, and the addition of oral antidiabetic agents or insulin therapy in some cases [4,5]. Traditionally, diabetes prevention and self-management education programs have been delivered in person with individual or face-to-face group interactions between health professionals and participants [6]. However, traditional in-person approaches have been hampered by low uptake and engagement rates [7]. Qualitative literature suggests that participants often find face-to-face programs difficult to attend because of issues with the timing of the courses, lack of transport, family and work commitments, or negative feelings toward participating in groups [8]. More recently, digital behavior change interventions (DBCIs) for diabetes prevention and management have emerged as potentially effective, scalable, and low-cost options to provide behavioral counseling when in-person programs are not accessible or attractive [9-11].

DBCIs are interventions that use digital technology to encourage and support behavior change that will maintain or improve health through the prevention and management of health problems and can, for example, be delivered through computer programs, websites, mobile apps, or wearable devices [12]. DBCIs may involve telehealth elements such as remote monitoring by health professionals who provide virtual support, either individually or in groups, or fully automated interventions that are based on algorithms [13]. DBCIs are becoming increasingly automated, interactive, and personalized because they use self-reports of users or sensor data to tailor feedback without the need for inputs from health professionals [14]. This development is facilitated by new technology-driven developments such as conversational agents (CAs) and just-in-time adaptive interventions (JITAIs). CAs, also known as chatbots, are computer systems that imitate human conversation using text or spoken language and can offer personalized human-like interactions [15-18]. Evidence from interventions using CAs show promising findings in terms of patient satisfaction [19], treatment success [20], and the capability to build work alliances with the patient [21-23]. CAs can also foster experiences equivalent to those offered by human coaches but with the additional advantage of being persistent and more consistent in providing choices that cultivate user autonomy [24]. This makes the use of CAs in DBCIs an encouraging component to complement or replace the need for human health professionals in intervention delivery.

Moreover, recent advances in wireless devices and mobile technology have enabled the design of JITAIs that can provide behavior change support at opportune moments and in response to an individual’s changing contexts [25-27]. More specifically, JITAIs adapt the provision of intervention content (eg, the type, timing, and intensity) by measuring the health condition or patient behavior with mobile technology such as smartphones, sensors, and software analytics to deliver intervention content at the time and in the context that the person needs it the most, and this is likely to improve health-related behaviors [25,27-29].

Novel technology-driven opportunities for DBCIs in diabetes care have attracted various health care stakeholders such as investors, health insurance companies, researchers, physicians, and patients [30]. The global market for digital diabetes care is rapidly growing and is expected to be worth US $1.5 billion in 2024 [31]. In 2018 alone, venture capital companies invested a record US $417 million into digital diabetes companies, a 12-fold increase in funding compared to 2013 [32]. However, little is known about the DBCIs provided by companies that have a substantial impact on the market, including the content of the interventions, how effective they are in managing and preventing type 2 diabetes, and the degree to which these interventions leverage new technology-driven developments such as CAs or JITAIs.

The aim of this paper is to systematically review the solutions provided by the top-funded companies offering DBCIs for type 2 diabetes prevention and management with a particular focus on how new technological developments, such as CAs and JITAIs, are being used to automate and scale-up intervention delivery. Therefore, the paper has the following objectives: (1) to identify the top-funded companies offering DBCIs for type 2 diabetes management and prevention, (2) to appraise the level of evidence to support these DBCIs in the form of peer-reviewed publications and recognition by national authorities for delivering evidence-based programs, and (3) to describe the characteristics of these DBCIs, with particular focus on the use of automation involved in the DBCIs by investigating the use of CAs, involvement of human health professionals, and what as well as how health and behavioral outcomes are measured that could be used to tailor JITAIs.

## Methods

### Searches

#### Companies

Digital health companies offering DBCIs were identified using 2 venture capital databases, Crunchbase Pro and Pitchbook [33,34]. Both databases are among the most comprehensive and accurate venture capital databases and are commonly used as data sources for academic reports and by investors [35]. We define digital health companies as companies that build and sell digital health products or services according to the definition of Safavi et al [36].

Searches were carried out on July 23, 2020, and they were updated on April 8, 2021 (Crunchbase Pro only). The total funding amount was last updated on June 15, 2021 using Crunchbase Pro). In case of conflicting funding information between the 2 databases, Crunchbase Pro data were reported, as Crunchbase Pro has better coverage than Pitchbook with respect to the financing rounds and total capital committed [35]. The search strategy included an extensive list of terms describing the constructs “verticals, methods, and industries,” “diabetes,” and “management and prevention.” The overview of the complete search strategy used for Crunchbase and Pitchbook is given in Table 1.

**Table 1:** Search strategy used in Crunchbase Pro and Pitchbook.

Search category	Search terms
1. Verticals, methods, and industries	Monitoring Equipment OR diagnostic OR HealthTech OR healthcare devices OR connected health* OR Therapeutic Devices OR Digital Health OR digital health* OR health* technology OR health* app* OR wearables OR Mobile health OR mhealth OR mobile app OR personal health OR virtual care OR e-health OR assistive technology OR telehealth OR telemedicine OR health* platform OR healthcare it OR data management OR Artificial Intelligence & Machine Learning OR Cloud data services OR analytics OR health* diagnostics OR Big Data OR information OR digital OR data OR biometrics OR home health care OR medtech OR self-monitoring
2. Diabetes	obesity OR blood sugar OR blood glucose OR insulin OR diabet*
3. Management and prevention	diabetes management OR diabetes treatment OR diabetes control OR diabetes monitoring OR blood sugar monitoring OR disease monitoring OR disease management OR risk reduction OR disease prevention OR diabetes prevention OR prevention OR prediabet*

#### Inclusion and Exclusion Criteria

We were interested in the companies having a substantial impact on the market and their ability to develop evidence-based solutions. Therefore, we decided to limit the scope of the analysis to the 15 top-funded companies defined as the leading companies in terms of the total funding amount, given that these companies are likely best equipped to develop and evaluate their interventions.

Companies were included if they (1) offered a DBCI for the prevention or management of type 2 diabetes and (2) involved a mobile app as the main intervention component. Companies were excluded if their DBCI (1) did not predominantly involve behavior change, (2) did not involve a mobile app as the main intervention component, and (3) did not focus on type 2 diabetes. We also excluded companies where the targeted conditions of the companies’ DBCIs were not clearly identifiable.

### Company Selection

Following the removal of duplicates, companies were ranked in the order of their funding amount. Company screening was conducted by screening from the most to the least funded companies until 15 companies eligible for inclusion in the study were identified. All the remaining companies were excluded due to insufficient funding amount. The list of the identified companies was reviewed by 3 experts with extensive industry and academic experience in the fields of digital health and type 2 diabetes to confirm that all relevant companies, covering the current market, had been identified through database searching. The experts included 2 scientific researchers with over 10 years of work experience with DBCIs at universities in the United Kingdom and United States and 1 industry expert with several years of work experience at one of the global market leaders for diabetes management systems in Germany.

### Publications

We searched PubMed and Google Scholar for scientific articles published up to April 30, 2021, using search terms “Name_Intervention” AND (Smartphone OR Application OR App OR Intervention OR Mobile Health) relating to the identified company’s DBCI. In addition, we identified studies by screening the websites of the companies for publication references.

#### Inclusion and Exclusion Criteria

To investigate the impact of the included DBCIs on health or behavioral outcomes in the study population, we included publications reporting quantitative results of experimental trials. Therefore, we excluded studies that did not involve effectiveness outcomes and those that did not report quantitative results. Furthermore, we excluded protocol studies and studies that targeted conditions other than type 2 diabetes.

### DBCIs

All the identified DBCIs included a mobile app as the main form of intervention delivery. We searched and downloaded all the identified apps from the 2 most popular app stores, Google Play Store and Apple App Store [37], between October 12, 2020, and April 10, 2021. If an app was not accessible, the companies were approached via email to request access. If no reply was received for the first email, a follow-up email was sent 2 weeks later. We also reviewed the DBCIs’ and companies’ websites as well as the identified publications for information on the characteristics of the DBCIs. Additional hardware devices such as activity trackers, blood glucose meters, wireless scales, or blood pressure devices that came as a part of the intervention program were not available and were therefore not reviewed.

### Data Extraction

Data extraction of companies, publications, and DBCIs was performed by 2 independent investigators (RK and SH). Disagreements were discussed and resolved by consensus. If no agreement was possible, disagreements were resolved through discussion with a third reviewer (GWT). Data extraction was performed using the Covidence Systematic Review software (Veritas Health Innovation Ltd) [38].

#### Companies

The extracted data for each company included the founding year, total funding amount, number of employees, and company headquarter location.

#### Publications

From the identified publications, we extracted the publication year, study design, number of participants, measured outcomes, quality of evidence (using the criteria of the US Preventive Services Task Force), journal impact factor, comparison to other treatment methods, and study findings. Similar to Safavi et al [36], the quality of individual studies was defined according to the USPSTF hierarchy of research design as follows: Level 1 includes evidence obtained from properly powered and conducted randomized controlled trials (RCTs), well-conducted systematic reviews, or meta-analyses of homogeneous RCTs. Level 2 includes evidence obtained from well-designed controlled trials without randomization, well-designed cohort or case-control analysis studies, or multiple time-series designs with or without the intervention or dramatic results in uncontrolled studies of large magnitude. Level 3 includes opinions of respected authorities, based on clinical experience or descriptive studies, or reports of expert committees [39]. As we were interested in the best available scientific evidence regarding the interventions, we extracted the results of publications with quality level 1. We specifically examined the primary outcome(s) from RCTs that were powered to detect change.

#### DBCIs

For each DBCI, we extracted the name of the intervention, name of the app, app accessibility information, number of app downloads (from Google Play Store only, as this information is not available on the Apple App Store), operating systems, cost, addressed category of the health care continuum (management or prevention), and the involvement of health professionals. For each DBCI with app access, we also extracted information on the availability of a CA and the measured health and behavioral outcomes. We were particularly interested in what and how health and behavioral outcomes were measured and if they could potentially be used to tailor JITAIs. Health and behavioral outcomes were defined as any biomarkers or health behaviors relevant for diabetes care such as diet, physical activity, or blood glucose tracking. Measurements included self-report data or sensor and device analytics [40-42]. More information on the framework used to assess the measurements of health and behavioral outcomes can be found in Multimedia Appendix 1.

We were also interested in whether the DBCIs were recognized by a national authority as an evidence-based program. For this purpose, we used the Diabetes Prevention Recognition Program (DPRP) developed by the US Centers for Disease Control and Prevention (CDC) [43]. The DPRP is the quality assurance arm of the National Diabetes Prevention Program, which is a partnership of public and private organizations that aim to prevent or delay type 2 diabetes [43]. Through the DPRP, the US CDC recognizes organizations that have demonstrated their ability to deliver an effective lifestyle change program. The organizations are required to use a CDC-approved curriculum and can deliver the intervention either in person by employing a trained human health coach or through a virtual setting with interaction involving a lifestyle coach [44]. The organizations are evaluated regularly based on the participant data submitted to the DPRP. These data need to fulfill a set of requirements, including a reduction in the risk of diabetes by achieving improvements in participant outcomes such as weight loss or glycated hemoglobin (HbA_1c_) reductions [44].

### Data Synthesis

The information extracted from the companies, publications, and DBCIs was summarized narratively.

## Results

### Selection and Inclusion of Companies

**Figure 1:**
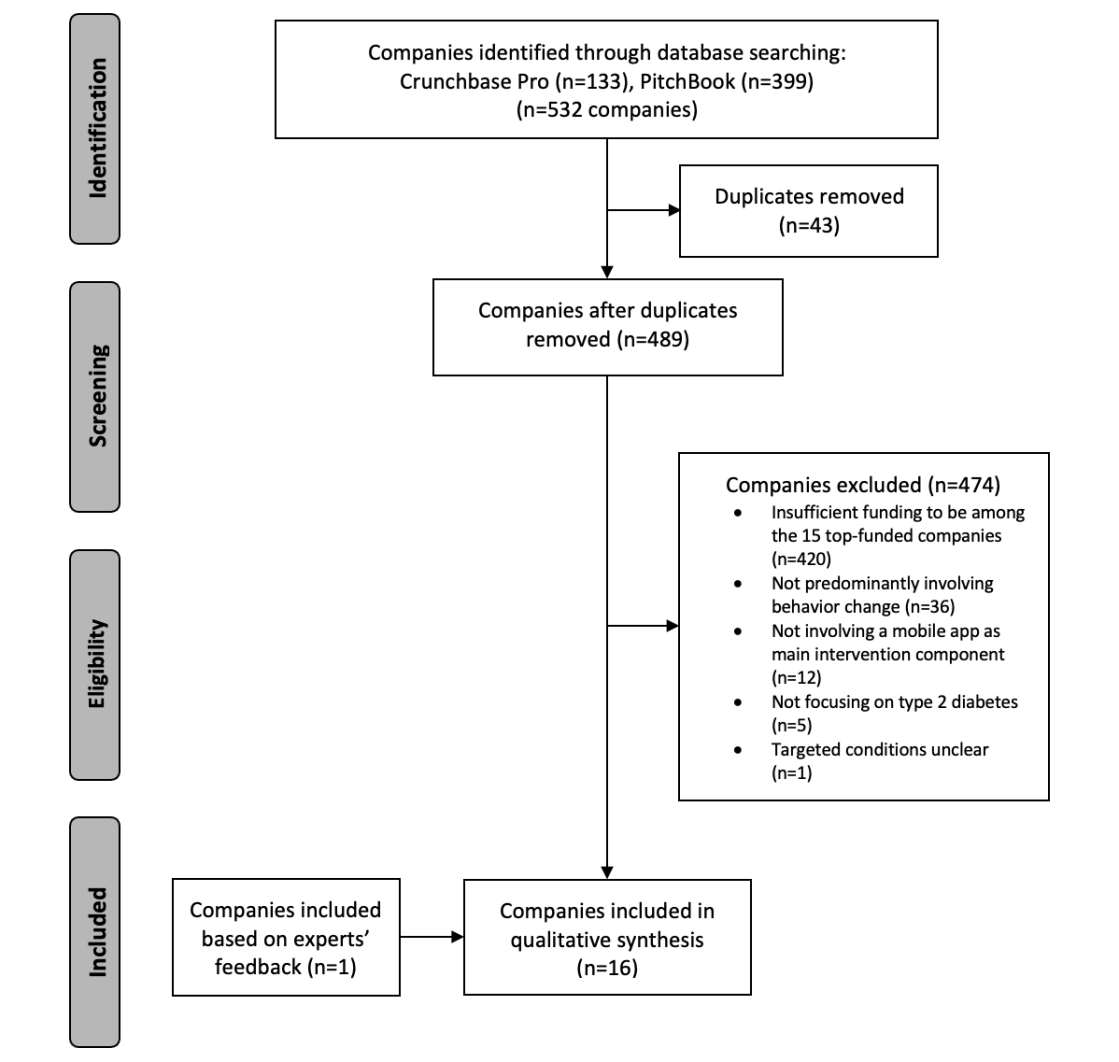
Flowchart of the company selection process.

The search yielded a total of 133 companies on Crunchbase Pro and 399 companies on Pitchbook. After removal of duplicates, 489 companies were eligible for screening. After screening, 54 companies were found to be ineligible for study inclusion, with the most common reason being not predominantly involving behavior change (36/54, 67%). Of the remaining 435 companies, 420 were excluded due to insufficient funding to be among the 15 top-funded companies. An additional company (KKT Technology Pte Ltd) was included on the recommendation of the independent experts, ultimately resulting in 16 companies eligible for study inclusion. Figure 1 outlines the selection process and reasons for exclusion. All the DBCIs of the included companies were available in English language.

The apps of 6 DBCIs were not accessible to the study authors (Virta, Dario, Welldoc, Liva, Twin, and Sweetch) because they were only available with a subscription service, in a specific geographic region, with an employer subscription, or when referred by a physician. Therefore, no information on the health and behavioral outcomes, measurements, or availability of CAs is provided on these apps within the results.

### Company Characteristics

The funding amount of the 16 top-funded digital health companies chosen for inclusion in the analysis ranged from US $657.3 to 15.5 million, totaling US $2.355 billion, as indicated in Table 2. Moreover, 11 companies (69%) were headquartered in the United States, 2 (13%) in the United Kingdom (13%), and 1 each in Denmark, Israel, and Singapore (6%). The year of founding ranged between 2005 and 2018, with 81% (13/16 companies) founded from 2011 onward. Additional information regarding the companies’ characteristics can be found in Multimedia Appendix 2.

**Table 2:** Overview of funding amounts determined for the included companies and scientific evidence obtained for the included digital behavior change interventions.

Company name	Funding (million US$)	DBCI^a^ name	Number of publications categorized by evidence level^b^			DPRP^c^ recognition^d^
			Level 1	Level 2	Level 3	
Noom Inc	657.3	Noom	1	7	0	Full
Virta Health Corp	373	Virta	0	7	0	None
Omada Health Inc	256.5	Omada	0	11	0	Full
Livongo Health Inc	235	Livongo	1	3	0	Full
Vida Health Inc	188	Vida	0	2	0	Full
DarioHealth Corp	169	Dario	0	0	0	None
Informed Data Systems Inc (One Drop)	106.2	One Drop	0	2	0	Pending
Lark Technologies Inc	95.7	Lark	0	1	0	Full
Welldoc Inc	55.2	BlueStar	1	5	0	Pending
Liva Healthcare ApS	43.5	Liva	1	3	0	None
Twin Health Inc	43.5	Twin	0	1	0	None
Oviva Inc	33	Oviva	0	2	0	None
KKT Technology Pte Ltd (Holmusk)	31.3	GlycoLeap	0	1	0	None
Sweetch Health Ltd	27.5	Sweetch	0	1	0	None
Nemaura Medical Inc	25	BEATdiabetes	0	0	0	None
Fruit Street Health Inc	15.5	Fruit Street	0	0	0	Full

^a^DBCI: digital behavior change intervention.

^b^Publication evidence level determined using the criteria of the US Preventive Services Task Force.

^c^DPRP: Diabetes Prevention Recognition Program.

^d^Recognition was established as none, pending, preliminary, or full, in line with the DPRP.

### Scientific Evidence

Totally 50 published studies related to the 16 companies’ DBCIs focusing on effectiveness were identified, as shown in Table 2. Further details on the study characteristics are available in Multimedia Appendix 3. The publication dates ranged from 2008 to 2021, with 86% (43/50) of the studies published from 2016 onward. The sample size of each study ranged from 16 to 35,921 participants. Out of the 50 studies, only 4 (8%) had quality level 1, evaluating DBCIs Noom, Livongo, BlueStar, and Liva. The remaining 46 studies (92%) had quality level 2. No studies were found for interventions Dario, BEATdiabetes, and Fruit Street. For 8 DBCIs, the recognition status in the DPRP of the US CDC was available, of which 6 DBCIs achieved full CDC recognition (Noom, Omada, Livongo, Vida, Lark, and Fruit Street), and 2 DBCIs had a pending recognition status (One Drop and BlueStar).

### Effectiveness of DBCIs

Of the 4 identified studies with quality level 1, 3 were RCTs having a duration of 12 months involving interventions Noom, BlueStar, and Liva [45-47], whereas 1 study involving Livongo [48] was a 6-month-long intervention tested within a randomized crossover trial spanning 12 months, with crossover at 6 months. BlueStar was the only intervention that resulted in a significantly greater improvement in the HbA_1c_ of the intervention group than that of the usual care group (mean difference 1.2%; 95% CI 0.5-1.9; *P*=.001) at 12 months follow-up [47]. In the study with Noom, Toro-Ramos et al [46] found no difference in the HbA_1c_ (mean difference 0.006%; SE 0.07; *P*=.93) between the intervention and control groups at 12 months follow-up [46]. Johansen et al [46] found that the Liva intervention did not reach the prespecified criterion for equivalence (mean difference −0.26%; 95% CI −0.52 to −0.01; *P*=.15) [46]. In the randomized crossover trial of Livongo, Amante et al [49] reported similar rates of HbA_1c_ change in both groups (intervention/usual care and usual care/intervention), and a significant treatment effect (mean change for intervention/usual care −1.1%, SD 1.5; mean change for usual care/intervention −0.8%, SD 1.5; *P*<.001) during the first 6 months. However, in the mixed-effects model, there was no significant improvement in HbA_1c_ between the intervention and usual care conditions (mean change 0.4%; *P*=.06). Compared to baseline, the interventions of Noom, Liva, and BlueStar showed HbA_1c_ reductions of 0.23% [45], 0.31% [46], and 1.9% [47] at 12 months, respectively. Using Livongo yielded HbA_1c_ reductions of 0.9% and 1.2% for the intervention/usual care and usual care/intervention group, respectively [48]. A summary of all the reported effectiveness measures among the identified scientific publications can be found in Multimedia Appendix 3.

### Characteristics of DBCIs

The full list of the included DBCIs is outlined in Table 3. Overall, 11 DBCIs were found to address diabetes prevention and management (Noom, Omada, Livongo, Vida, Lark, BlueStar, Liva, Oviva, GlycoLeap, Sweetch, and BEATdiabetes), whereas 4 DBCIs addressed only diabetes management (Virta, Dario, One Drop, and Twin), and 1 solely focused on diabetes prevention (Fruit Street). The program costs varied, ranging from US $19.99 to $249 per month, whereas some were available on an annual basis or covered by health care providers, health plans, or employers. Furthermore, 11 DBCIs (Noom, Virta, Omada, Vida, One Drop, BlueStar, Liva, Oviva, GlycoLeap, BEATdiabetes, and Fruit Street) involved a human health professional as part of the intervention delivery, and 2 DBCIs (Livongo and Dario) offered it as an optional feature. Among the 3 remaining DBCIs, 2 did not employ a health professional (Lark and Sweetch), and this could not be determined in 1 DBCI (Twin). Of the 16 included DBCIs, 10 apps were accessible to the authors. Only 1 of the 10 accessible apps employed a CA (Lark).

We found that all the 10 accessible apps (10/16, 63%) tracked health or behavioral outcomes using self-reports as well as sensor and device analytics. Diet and body weight were the most frequently tracked health and behavioral outcomes (n=10), followed by physical activity or exercise (n=9), blood glucose (n=7), blood pressure and HbA_1c_ (n=5), mood (n=3), sleep (n=3), medication (n=2), waist circumference (n=1), well-being (n=1), calories (n=1), heart rate (n=1), and stress (n=1).

**Table 3:** Intervention delivery characteristics of the companies’ digital behavior change interventions.

DBCI^a^ name	Health continuum category	Cost	HHP^b^ involved	CA^c^ used	Tracked health and behavioral outcomes	Self-reports; sensor and device analytics
Noom	Prevention and management	US $59/month or $199/year	Yes	No	Physical activity, body weight, sleep, diet, and blood pressure	Open questions, ratings, multiple choice, physical activity recordings, and accelerometer gyroscope
Virta	Management	US $249/month plus a one-time $250 initiation fee	Yes	—^d^	—	—
Omada	Prevention and management	US $140/month for the first 4 months and $20/month for the following months	Yes	No	Blood glucose, physical activity, body weight, diet, and blood pressure	Open questions, ratings, multiple choice, body sensors, physical activity recordings, and Bluetooth
Livongo	Prevention and management	Purchase free; costs covered by employer, health plan, or health care provider	Yes, but optional	No	HbA_1c_^e^, blood glucose, physical activity, body weight, diet, and blood pressure	Open questions, ratings, body sensors, camera, Bluetooth, and accelerometer gyroscope
Vida	Prevention and management	Free download, free 1 week trial, and subscription US $58.25-$79/month	Yes	No	HbA_1c_, physical activity, body weight, stress, and diet	Open questions, ratings, multiple choice, and Bluetooth
Dario	Management	Basic US $25-$30/month, pro US $33-$40/month, and premium US $70-$85/ month	Yes, but optional	—	—	—
One Drop	Management	Digital membership US $19.99/month, supplies $20.99/month, and combined package $30.99/month	Yes	No	HbA_1c_, blood glucose, physical activity, body weight, medication, diet, and blood pressure	Open questions, ratings, multiple choice, location, camera, and telephone
Lark	Prevention and management	Lark Weight Loss Pro US $19.99, Lark Wellness Pro $14.99, and Lark Diabetes Prevention Program Pro $119.99	No	Yes	Physical activity, body weight, sleep, mood, well-being, and diet	Open questions, ratings, multiple choice, Bluetooth, accelerometer gyroscope, GPS^f^, and app usage
BlueStar	Prevention and management	Unclear	Yes	—	—	—
Liva	Prevention, Management	Unclear	Yes	—	—	—
Twin	Management	INR^g^ 1450 (1 INR=US $0.01344) for a 14-day trial; price for continuous use unclear	Unclear	—	—	—
Oviva	Prevention and management	CHF^h^ 484 (1 CHF=US $1.09204) carried by health care provider	Yes	No	Blood glucose, physical activity, body weight, mood, and diet	Open questions, ratings, multiple choice, and camera
GlycoLeap	Prevention and management	Free, but only available for diabetic and prediabetic patients through their doctor if they are part of the project or through particular employers	Yes	No	HbA_1c_, blood glucose, body weight, mood, and diet	Open questions, ratings, camera, Bluetooth, and photos
Sweetch	Prevention and management	Unclear	No	—	—	—
BEATdiabetes	Prevention and Management	Unclear	Yes	No	HbA_1c_, blood glucose, physical activity, body weight, medication, waist circumference, and diet	Open questions, ratings, multiple choice, and Bluetooth
Fruit Street	Prevention	US $19.99/month	Yes	No	Blood glucose, physical activity, body weight, sleep, heart rate, calories, diet, and blood pressure	Open questions, ratings, physical activity recordings, camera, and Bluetooth

^a^DBCI: digital behavior change intervention.

^b^HHP: human health professional.

^c^CA: conversational agent.

^d^—app not accessible.

^e^HbA_1c_: glycated hemoglobin A_1c_.

^f^GPS: Global Positioning System.

^g^INR: Indian Rupee.

^h^CHF: Swiss Franc.

**Figure 2:**
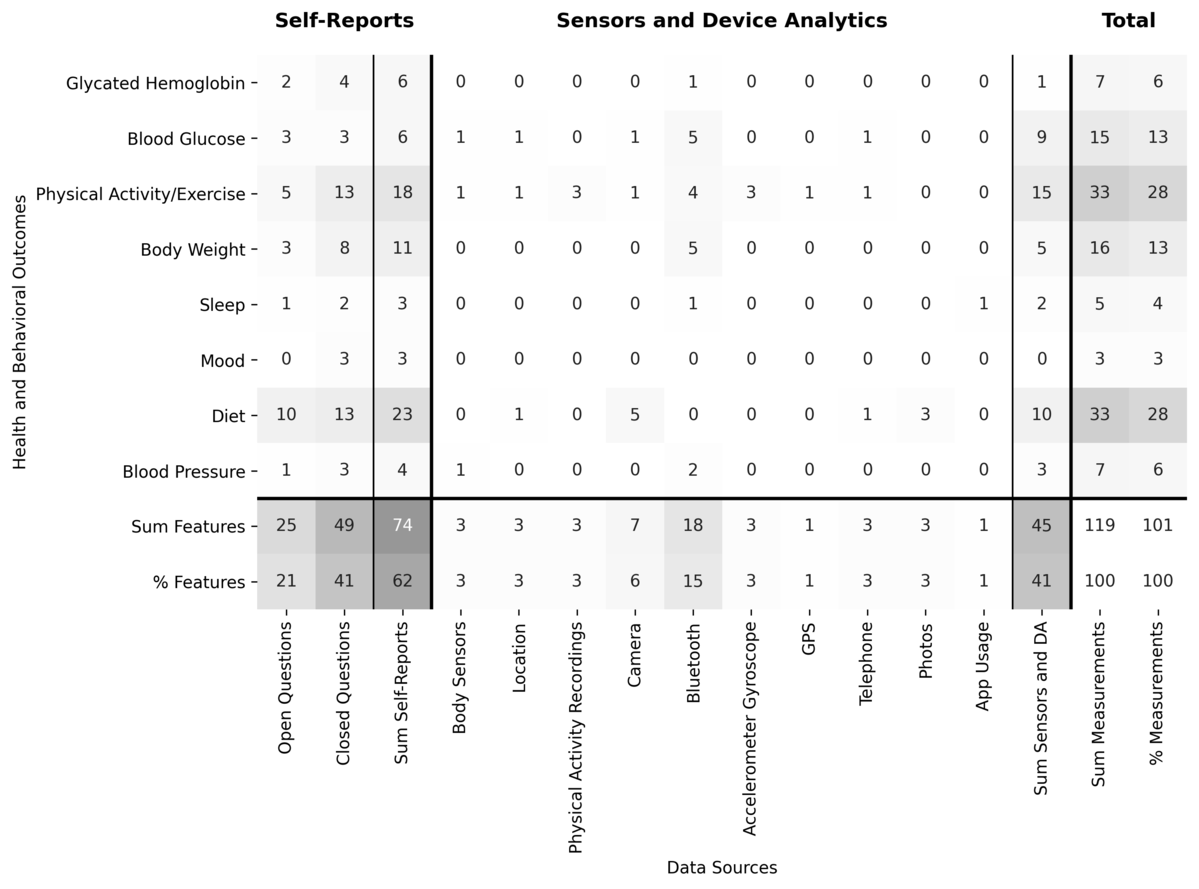
Gray scale illustrating the number of times health or behavioral outcomes were measured by self-reports or sensor and device analytics summarized considering all the 10 reviewed apps. DA: device analytics; GPS: Global Positioning System.

The findings regarding the usage of self-reports as well as sensor and device analytics are summarized in Figure 2. In the 119 usages considered, self-reports were used 74 times (62%), whereas sensor and device analytics were used 45 times (38%) as the data source of the 10 accessible apps. Self-reports were most frequently measured by closed questions including ratings, Likert scales, and multiple-choice questions (49 times, 41%) followed by open questions (25 times, 21%). The sensor and device analytics that were most frequently used were Bluetooth and cameras, which were used 18 (15%) and 7 times (6%), respectively. The darker color indicates a higher number of occurrences.

## Discussion

### Principal Results

Of the 16 companies and DBCIs included in this review, only 4 were assessed for their effectiveness in changing HbA_1c_ via high-quality RCTs. Results from the 4 RCTs analyzed indicate these DBCIs have a varying effect on HbA_1c_. For example, the BlueStar intervention showed a significant improvement of 1.2% in HbA_1c_ compared to the usual care group at 12 months, whereas the Noom, Livongo, and Liva interventions did not show any significant improvements. Furthermore, there was a wide range in the number of effectiveness studies across DBCIs, with 1 study having no published scientific evidence to 1 having 11 associated publications. We found a trend toward more published studies involving higher-funded companies, with the 3 top-funded companies (Noom, Virta, and Omada) accounting for more than half (26/50, 52%) of all publications. We also found that 5 of the highest-funded DBCIs achieved full recognition status from the DPRP (Noom, Omada, Livongo, Vida, and Lark), whereas only 1 among the lower-funded companies with funding ranks 9 to 16 (Fruit Street) received full DPRP recognition. Further, 2 DBCIs in our sample (Dario and BEATdiabetes) were neither recognized by the DPRP nor had any published effectiveness studies available. More adequately powered and high-quality RCTs are needed to confirm the effectiveness of top-funded DBCIs for type 2 diabetes prevention and management.

Recognition by national authorities to deliver evidence-based programs can be an important reference point for potential consumers and physicians when deciding to use or prescribe a particular intervention program and can serve to incentivize the adoption of impact-focused interventions [36]. Recognition can benefit the companies offering the interventions by providing sustainability and reimbursement for the intervention through many private and public payers that require recognition, such as Medicare [49]. Recognition can also be an effective marketing tool and encourage referrals. However, we only found 1 certification program for evidence-based diabetes prevention or management programs, which was the DPRP offered by the US CDC [43]. This lack of quality assurance programs could hamper consumers’ and health care providers’ decision-making processes when identifying the most effective programs. Therefore, additional quality assurance programs that can certify diabetes prevention and management interventions based on evidence-based criteria are necessary, especially for diabetes management interventions and in countries other than the United States.

Reduction in HbA_1c_ is one of the key clinical outcomes for assessing the effectiveness of interventions for type 2 diabetes prevention and management and is also one of the effectiveness criteria to achieve recognition by the DPRP [44]. In the 4 RCTs evaluated in our analysis, the Noom and Liva interventions showed modest HbA_1c_ reductions of 0.2% to 0.3% [45,46], whereas the BlueStar and Livongo interventions showed higher HbA_1c_ reductions of over 1% [47,48]. According to the criteria of the DPRP, an HbA_1c_ reduction of 0.2 percentage points is considered sufficient for a lifestyle change program to receive recognition [44], although a change of 0.4% to 0.5% is considered a clinically meaningful improvement [50]. The 4 RCTs reviewed [46-49] were also powered to detect changes in HbA_1c_ between 0.4% and 1%. Therefore, this raises the question of whether the effectiveness criterion of the DPRP standards around the change in HbA_1c_ is sufficient. Furthermore, even though recognition from the DPRP guarantees that a certain level of diabetes risk reduction was achieved because of a specific DBCI, the recognition does not give any further information on the magnitude of the reduction, as data that companies submit to achieve DPRP recognition are not made publicly available. This lack of information limits transparency for researchers, investors, users, and payers to identify the most effective programs. Moreover, this lack of data transparency could become even more troublesome if companies that are already recognized to deliver evidence-based programs are then unwilling to invest additional resources into research and development. Therefore, we highlight the need for more transparency regarding data related to the effectiveness of DBCIs. We believe that our findings also indicate the importance of encouraging the digital health industry to build more evidence-based DBCIs. Clarifying the regulatory landscape around DBCIs and developing incentives that lead to a stronger customer market have been identified as 2 possible areas that policy makers may address to foster such an encouragement [36]. In addition, we recognize the poor standard of reporting by the DBCI companies regarding the app features, employed behavior change techniques, and information on what and how sensing data are being utilized. This lack of transparent reporting is likely because companies that develop these proprietary apps tend to be reluctant to disclose app details that could potentially be useful for competitors. From a research perspective, this lack of transparency makes it difficult to compare intervention features objectively. It also reveals the need for more transparent reporting on the characteristics of DBCIs by the companies.

In our reviewed DBCIs, the most commonly tracked health and behavioral outcomes were diet and body weight, which were tracked in all the 10 accessible apps, followed by physical activity or exercise, which was tracked in 9 apps. Other frequently tracked outcomes were blood glucose (7 apps), blood pressure, and HbA_1c_ (5 apps each). Our findings are in line with previous studies that reviewed apps for self-management and lifestyle modification in type 2 diabetes patients [51-53] and are also similar to the opinion of clinical experts regarding important intervention components [53,54]. However, we found that less than 40% of health and behavioral outcomes were measured using sensors and device analytics and that most outcomes were measured by self-reports. Although such self-reports can be used in the form of ecological momentary assessments [55] that are closely related to the concept of JITAIs [56], self-reports can be burdensome for participants to complete and may lead to difficulties in keeping users engaged [25,57]. Therefore, we believe that self-reports are not sufficient to leverage the full potential of JITAIs. The low usage of measurements from sensor and device analytics indicates that it is unlikely that the investigated interventions use JITAI mechanisms to tailor the intervention content to the user. In addition, there is no clear evidence on how these intervention components are related to intervention effectiveness; therefore, future studies must identify which DBCI features most successfully impact intervention effectiveness.

Our review also aimed to assess the extent to which human health professionals and automated CAs are used within the DBCIs. We found that 13 of the 16 DBCIs involved a human health professional, of which 2 DBCIs offered it as an optional feature. We found that among the 10 apps that were available to us, only 1 app used a CA. The high usage of human health coaches alongside the low usage of CAs, and the unlikely use of JITAI mechanisms to tailor intervention content, indicates the low use of automation among the investigated DBCIs. This limits the overall scalability of existing DBCIs and the potential of the interventions to reach a greater proportion of the eligible population [58] because the involvement of human health coaches is generally time- and resource-intensive.

We identified 4 potential reasons that might account for this low use of automation among the investigated DBCIs. First, automated approaches, such as CAs, are still part of an emerging area within type 2 diabetes management and DBCIs. It is possible that users might have concerns when relying on CAs for actionable medical information around diabetes [59]. Second, app features that use sensor technologies might not be adequately developed to replace input from human health professionals or self-report methods, thus leading to significant user burden. For example, the current state-of-the-art food volume estimation approaches to assess dietary intake are not yet usable in commercial apps due to several gaps and technological issues [60]. Therefore, many apps rely on user inputs, for example, by selecting serving sizes of identified foods, based on which nutritional values can be estimated [60]. Third, there appears to be insufficient evidence to support the widespread use of fully automated approaches without remote access to a human health professional [13]. Thus, additional RCTs or cohort studies that directly compare DBCIs involving digital human coaches with fully automated approaches are needed to better understand the potential and effectiveness of automated DBCIs. Fourth, in the current standards and operating procedures of the DPRP, live interactions with lifestyle coaches should be offered at least on a weekly basis during the first 6 months [44]. Although email and text message interactions may contribute toward this requirement, it is likely to be challenging for companies aiming to offer fully automated DBCIs to meet this requirement. Recognition by the DPRP is valuable to many companies [49]; nevertheless, satisfying the requirement of offering live coaching interactions prevents the recognition of fully automated approaches and limits the scalability of DBCIs for type 2 diabetes prevention. Further research is warranted to establish if human coaches are indeed necessary to deliver an effective lifestyle change program.

### Strengths and Limitations

This study has several strengths. First, we conducted a comprehensive company search involving 2 widely used venture capital databases [35], and we had 3 independent digital health experts confirm that the final list of included companies covered the market. Second, we conducted comprehensive data extraction using multiple sources, including databases, intervention websites, peer-reviewed publications, and mobile apps. Third, we summarized only the highest quality scientific evidence on the effectiveness of the included DBCIs.

However, our review has some limitations. First, even though we identified the top-funded companies in the field, this does not guarantee that their interventions reach a significant proportion of the target population. Many of the reviewed companies are still in the start-up phase where they typically acquire considerable funding; however, their DBCIs may have limited accessibility, for example, only through referral by partnering clinicians. Second, we were only able to access 10 out of the 16 DBCI apps, as some apps were only accessible with a subscription service, in a specific geographic region, with a doctor’s prescription, an access code, or through an employer subscription. Although we systematically contacted the companies and requested app access, we only received additional access to 5 paid or proprietary apps through the companies. Third, we were unable to access, and therefore assess, any additional devices that may have accompanied the DBCI apps. Some of these devices record additional health parameters via sensors, such as (smart) blood glucose meters, (smart or wireless) scales, activity trackers, or smartwatches. Therefore, we could not assess all the functionalities of these devices, which limited the comprehensiveness of our review. Fourth, we were not able to assess certain app features that were behind a paywall. This was often the case for support that was delivered through health professionals. Fifth, the DPRP is only relevant for interventions targeting diabetes prevention and therefore does not cover DBCIs that solely target diabetes management. In addition, not all reviewed DBCIs were available in the United States; consequently, they are not eligible to achieve recognition by the DPRP. Sixth, given that most of the investigated DBCIs and all DBCIs with a corresponding fully powered RCT address diabetes management and diabetes prevention, it was not feasible to separately report the results in these 2 categories.

### Comparison With Prior Work

This is the first systematic assessment of the top-funded companies that offer DBCIs for type 2 diabetes prevention or management. Previous reviews have focused on apps and digital interventions for diabetes management, but they were mostly limited to interventions reported in scientific research without a particular impact on the market [51,52,61-64]. These reviews generally found DBCIs to be effective in improving diabetes-related outcomes, particularly HbA_1c_ [51,52,61-64], which is in line with our findings; nevertheless, they also concur that the current evidence is limited and there is a need for adequately powered, rigorous trials with long-term follow-ups to determine the clinical and economic impact of such interventions [52,65]. In terms of JITAIs, a recent systematic review investigating popular mental health apps for individuals with depression concluded that JITAI mechanisms have not yet been translated into mainstream depression apps [66], which also aligns with our findings.

### Conclusions

Our findings suggest that the level of funding received by companies offering DBCIs for type 2 diabetes prevention and management does not coincide with the level of evidence on the intervention effectiveness. There is significant variation in the level of evidence underpinning the different DBCIs and an overall need for more rigorous effectiveness trials as well as additional certification programs for evidence-based diabetes prevention and management interventions in countries other than the United States. In addition, we emphasize the need for more data transparency from quality assurance authorities to inform stakeholders and consumers on how effective each DBCI is in improving diabetes-related outcomes. We further found low usage of CAs, an unlikely use of JITAI mechanisms, and a high level of support from human health professionals among the apps investigated, which indicates low usage of automated approaches. Because automation and technology are critical factors to increase the interventions’ scalability, further research is warranted to establish the effectiveness of fully automated DBCIs in comparison to those offering support from human health professionals. Finally, we recommend that national authorities such as the DPRP help reduce barriers for the recognition of fully automated approaches and encourage policy makers to foster an environment that encourages the digital health industry to build more evidence-based solutions.

## Appendix

Multimedia Appendix 1 Codebook for app review.

Multimedia Appendix 2 Company and intervention characteristics.

Multimedia Appendix 3 Study characteristics.

## Abbreviations

CAs: conversational agents
CDC: Center for Disease Control and Prevention
DBCIs: digital behavior change interventions
DPRP: Diabetes Prevention Recognition Program
HbA_1c_: glycated hemoglobin A1c
JTAI: just-in-time adaptive intervention
RCTs: randomized controlled trials
