# Supplementary material for: Digital Behavior Change Interventions for the Prevention and Management of Type 2 Diabetes: Systematic Market Analysis
Source: J Med Internet Res. 2022 Jan 7;24(1):e33348. doi: 10.2196/33348 (PMC8783286; doi:10.2196/33348)
Supplement: Multimedia Appendix 1 [file jmir_v24i1e33348_app1.docx]

# Supplementary Files

**Supplementary Table 1. Codes for the classification of self-reports.**

| **Code** | **Self-Reports** | **Source** | **Description** |
| --- | --- | --- | --- |
| SR1 | Open Questions | ·· | Questions with non-restricted input |
| SR2 | Ratings | ·· | Questions answered with a scale (e.g. Likert Scale) |
| SR3 | Multiple Choice | ·· | Question with a given number of answer options |

**Supplementary Table 2. Codes for the classification of sensors.**

| **Code** | **Sensor** | **Source** | **Description** |
| --- | --- | --- | --- |
| PS1 | Vital Signs | Android Developers Guide (2020)[1] | The app can access sensor data about vital signs |
| PS2 | Location | Android Developers Guide (2020)[1] | The app can access the device's location |
| PS3 | Physical Activity Recordings | Android Developers Guide (2020)[1] | The app can access recordings of physical activity, such as walking, cycling, driving, step count, etc. |
| PS4 | Camera | Android Developers Guide (2020)[1], iOS Security Guide(2020)[2] | The app can take pictures and record video |
| PS5 | Microphone | Android Developers Guide (2020)[1], iOS Security Guide (2020)[2] | The app can record audio |
| PS6 | Speech recognition | Android Developers Guide (2020)[1], iOS Security Guide (2020)[2] | The app can use speech recognition |
| PS7 | Bluetooth | Android Developers Guide (2020)[1], iOS Security Guide (2020)[2], Cornet et al (2018)[3] | The app can detect or communicate with other Bluetooth-enabled devices |
| PS8 | Accelerometer | Cornet et al (2018)[3] | The app can measure the speed of movement in space and the speed of the rotation of the device |
| PS9 | GPS | Cornet et al (2018)[3] | The app can use the information of four or more GPS satellites to calculate the position of the device |
| PS10 | Antenna | Cornet et al (2018)[3] | The app can use the information of nearby cellular towers and relay the single to the broadband processor for voice/SMS/ data communication |
| PS11 | Light Sensor | Cornet et al (2018)[3] | The app can measure the amount of light reaching the device |
| PS12 | Proximity | Cornet et al (2018)[3] | The app can detect the proximity between the front of the phone and any obstacle, such as a human face |

**Supplementary Table 3. Codes for the classification of device analytics.**

| **Code** | **Device Analytics** | **Source** | **Description** |
| --- | --- | --- | --- |
| DA1 | Storage | Android Developers Guide (2020)[1] | The app can access photos, media, and files on the device |
| DA2 | Telephone | Android Developers Guide (2020)[1] | The app can make and manage phone calls |
| DA3 | Photos | Android Developers Guide (2020)[1] | The app can access the photos stored |
| DA4 | Contacts | Android Developers Guide (2020)[1], iOS Security Guide (2020)[2] | The app can access the phone contacts |
| DA5 | Call logs | Android Developers Guide (2020)[1], Cornet et al (2018)[3] | The app can access the number of times a call was made, accepted, and missed |
| DA6 | Calendar | Android Developers Guide (2020)[1], iOS Security Guide (2020)[2] | The app can access the calendar |
| DA7 | Device activity | Cornet et al (2018)[3] | The app can access recorded activity time of the device |
| DA8 | SMS patterns | Android Developers Guide (2020)[1], Cornet et al (2018)[3] | The app can access the number of messages send and received |
| DA9 | Application usage | Cornet et al (2018)[3] | The app can access the apps used and times the apps were used |
| DA10 | Browser history | Cornet et al (2018)[3] | The app can access visited websites |

## References

1. Android Sensors Overview [database on the Internet]. 2020 [cited 2020 July 07]. Available from: https://developer.android.com/guide/topics/sensors/sensors_overview.

2. iOS Security Guide [database on the Internet]. 2020 [cited 2020 July 07]. Available from: https://support.apple.com/en-gb/guide/security/welcome/web.

3. Cornet VP, Holden RJ. Systematic review of smartphone-based passive sensing for health and wellbeing. J Biomed Inform. 2018 Jan;77:120-32. PMID: 29248628. doi: 10.1016/j.jbi.2017.12.008.
