# Supplementary material for: Digital Behavior Change Interventions for the Prevention and Management of Type 2 Diabetes: Systematic Market Analysis
Source: J Med Internet Res. 2022 Jan 7;24(1):e33348. doi: 10.2196/33348 (PMC8783286; doi:10.2196/33348)
Supplement: Multimedia Appendix 2 [file jmir_v24i1e33348_app2.pdf]

Aspect 1: Company characteristics

Company name	Headquarter location	Year founded	Employees	Funding (million USD)
Noom, Inc.	New York, NY	2008	1001-5000	657.3
Virta Health Corp.	San Francisco, CA	2014	251-500	373.0
Omada Health, Inc.	San Francisco, CA	2011	251-500	256.5
Livongo Health, Inc.	Mountain View, CA	2008	251-500	235.0
Vida Health, Inc.	San Francisco, CA	2014	501-1000	188.0
DarioHealth Corp.	New York, NY	2011	11-50	169.0
Informed Data Systems, Inc. (One Drop)	New York, NY	2014	1-10	106.2
Lark Technologies, Inc.	Mountain View, CA	2011	101-250	95.7
Welldoc, Inc.	Columbia, MD	2005	101-250	55.2
Liva Healthcare ApS	Copenhagen, Hovedstaden, Denmark	2014	11-50	43.5
Twin Health, Inc.	Mountain View, California, United States	2018	101-250	43.5
Oviva, Inc.	London, England, United Kingdom	2013	101-250	33.0
KKT Technology Pte. Ltd. (Holmusk)	Singapore, Central Region, Singapore	2015	11-50	31.3
Sweetch Health Ltd.	Jerusalem, Yerushalayim, Israel	2013	1-10	27.5
Nemaura Medical Inc.	Loughborough, Leicestershire, United Kingdom	2011	1-10	25.0
Fruit Street Health P.B.C.	New York, NY	2014	101-250	15.5

Aspect 2: Scientific evidence

DBCI	Number of publications categorised by evidence level			Number of participants over all studies	Average journal impact factor	DPRP recognition status
	Level 1	Level 2	Level 3			
Noom	1	7	0	51367	3.7	Full
Virta	0	7	0	1755	4.9	No
Omada	0	11	0	17388	3.3	Full
Livongo	1	3	0	5079	4.3	Full
Vida	0	2	0	1695	3.5	Full
Dario	0	0	0	NA	NA	No
One Drop	0	2	0	1544	4.4	Pending
Lark	0	1	0	70	4.5	Full
BlueStar	1	5	0	607	6.1	Pending
Liva	1	3	0	294	13.7	No
Twin	0	1	0	64	3.2	No
Oviva	0	2	0	212	4.3	No
GlycoLeap	0	1	0	100	4.3	No
Sweetch	0	1	0	55	4.9	No
BEATdiabetes	0	0	0	NA	NA	No
Fruit Street	0	0	0	NA	NA	Full

Abbreviations: DBCI=Digital behaviour change intervention, DPRP=Diabetes Prevention Recognition Program by the US Centers for Disease Control and Prevention, NA=No studies available

Aspect 3: Characteristics of DBCIs

Name of app	Name of the intervention Program	App access-ible	Tracked health and behavioural outcomes	Self-Reports used	Sensor and Device Analytics used	Operating systems	Number of downloads (Google Play Store)	Cost	Health continuum category	Conver-sational agent used?	Human health profess-ionals involved	Additional devices as part of the intervention program
Noom	Noom program	Yes	Physical activity, Body weight, Sleep, Diet, Blood pressure	Open questions, Ratings, Multiple choice	Physical activity, Accelerometer gyroscope	iOS, Android	10000000+	$59.00 USD/month or $199.00 USD/year	Prevention, Management	No	Yes	NA
Virta Health	Virta Program	No	-	-	-	iOS, Android	1000+	Unclear	Management	-	Yes	Starter kit with wireless body weight scale, blood glucose and ketone meter and testing strips, lancets, protein food scale, blood pressure cuff (optional)
Omada	Omada Health Program	Yes	Blood glucose, Physical activity, Body weight, Diet, Blood pressure	Open questions, Ratings, Multiple choice	Body sensors, Physical activity, Bluetooth	iOS, Android	100000+	$140/month for the first 4 months, $20/month for following months.	Prevention, Management	No	Yes	Wireless scale, pedometer, tape for measurement (waist), resistance band
Livongo	Livongo for Diabetes Program	Yes	HbA1c, Blood Glucose, Physical activity, Body weight, Diet, Blood pressure	Open questions, Ratings	Body sensors, Camera, Bluetooth, Accelerometer gyroscope	iOS, Android	100000+	Purchase free, costs covered by employer or healthcare provider	Prevention, Management	No	Yes, but optional	Livongo blood glucose meter and all necessary materials (lancet, test stripes) and transport case.
Vida Health	Vida Health Diabetes Prevention Program	Yes	HbA1c, Physical activity, Body weight, Stress, Diet,	Open questions, Ratings, Multiple choice	Bluetooth	iOS, Android	10000+	Free download, free 1 week trial, subscription $58.25-$79/month	Prevention, Management	No	Yes	NA

Name of app	Name of the intervention Program	App access-ible	Tracked health and behavioural outcomes	Self-Reports used	Sensor and Device Analytics used	Operating systems	Number of downloads (Google Play Store)	Cost	Health continuum category	Conver-sational agent used?	Human health profess-ionals involved	Additional devices as part of the intervention program
Dario Health	Unclear	No	-	-	-	iOS, Android	100000+	Basic $25-$30/month, pro $33-$40/month, premium $70-$85/ month	Management	-	Yes, but optional	Dario Health all-in-one device (with lancing device, blood glucose meter and placeholder for strips)
One Drop: Transform Your Life	One Drop Program	Yes	HbA1c, Blood glucose, Physical activity, Body weight, Medication tracking, Diet, Blood pressure	Open questions, Ratings, Multiple choice	Location, Camera, Telephone	iOS, Android	1000000+	Digital membership $19.99/month, supplies $20.99/month, combined package $30.99/month	Management	No	Yes	One Drop blood glucose meter (optional; for 20$/month with unlimited testing strips)
Lark	Lark Diabetes Prevention Program	Yes	Physical activity, Body weight, Sleep, Mood, Wellbeing, Diet	Open questions, Ratings, Multiple Choice	Bluetooth, Accelerometer gyroscope, GPS, Application usage	iOS, Android	100000+	Lark Weight Loss Pro $19.99, Lark Wellness Pro $14.99, Lark Diabetes Prevention Program Pro $119.99	Prevention, Management	Yes	No	Preventive insurance benefit for prediabetes covers a Lark blood sugar meter, Lark scale, Fitbit
BlueStar Diabetes	Unclear	No	-	-	-	iOS, Android	10000+	Unclear	Prevention, Management	-	Yes	Unclear
Liva UK	Unclear	No	-	-	-	iOS, Android	10000+	Unclear	Prevention, Management	-	Yes	NA

Name of app	Name of the intervention Program	App access-ible	Tracked health and behavioural outcomes	Self-Reports used	Sensor and Device Analytics used	Operating systems	Number of downloads (Google Play Store)	Cost	Health continuum category	Conver-sational agent used?	Human health profess-ionals involved	Additional devices as part of the intervention program
Twin Health	Twin Precision Nutrition Program	No	-	-	-	iOS, Android	5000+	1450 Rs (ca. 19.80 USD) for a 14 days trial, price for continuous use unclear	Management	-	Unclear	Abbott Freestyle Libre Pro GCM sensor
Oviva	Oviva Program	Yes	Blood glucose, Physical activity, Body weight, Mood, Diet	Open questions, Ratings, Multiple choice	Camera	iOS, Android	10000+	CHF 484 carried by healthcare provider	Prevention, Management	No	Yes	NA
GlycoLeap - Your Health Coach	GlycoLeap	Yes	HbA1c, Blood glucose, Body weight, Mood, Diet	Open questions, Ratings	Camera, Bluetooth, Photos	iOS, Android	1000+	Free, but only available for diabetic and prediabetic patients through their doctor if they are part of the project or through particular employers	Prevention, Management	No	Yes	Glucometer, refills of test strips, fitness tracker, connected weighing scale
Sweetch Health	Unclear	No	-	-	-	iOS, Android	500+	Unclear	Prevention, Management	-	No	Unclear
BEATdiabetes	BEATdiabetes	Yes	HbA1c, Blood Glucose, Physical activity, Body weight, Medication tracking, Waist circumference, Diet	Open questions, Ratings, Multiple choice	Bluetooth	iOS, Android	5+	Unclear	Prevention, Management	No	Yes	Unclear
Fruit Street	Fruit Street's Diabetes Prevention and Weight Loss Program	Yes	Blood glucose, Physical activity, Body weight, Sleep, Heart rate, Calories, Diet, Blood pressure	Open questions, Ratings	Physical activity, Camera, Bluetooth	iOS, Android	1000+	$19.99/month	Prevention	No	Yes	Wireless scale & Fitbit activity tracker

Abbreviations: -=App not accessible, DBCI=Digital behaviour change intervention, NA=No additional devices included
