# Supplementary material for: Digital Behavior Change Interventions for the Prevention and Management of Type 2 Diabetes: Systematic Market Analysis
Source: J Med Internet Res. 2022 Jan 7;24(1):e33348. doi: 10.2196/33348 (PMC8783286; doi:10.2196/33348)
Supplement: Multimedia Appendix 3 [file jmir_v24i1e33348_app3.pdf]

DBCI	Study authors name	Study title	Year of publication	Journal impact factor	Design	Number of participants	Outcomes measured	Comparison to other treatment	Findings of RCTs
Noom	Ku, E.J.; Park, J.; Jeon, H.J.; Oh T.; Choi, H.J.	Clinical Efficacy and Plausibility of a Smartphone-based Integrated Online Real-time Diabetes Care System via Glucose and Diet Data Management: A Pilot Study	2020	1.767	RCT	40	HbA1c*; Self-care	Yes, compared to conventional care	#not adequately powered
Noom	Kim, Y.; Oh, B.; Shin, H.	Effect of mHealth With Offline Antiobesity Treatment in a Community-Based Weight Management Program: Cross-Sectional Study	2020	4.301	NCT	150	Weight; BMI; Waist circumference; Fat mass; Metabolic equivalent	No, no comparison to other treatment	-
Noom	Toro-Ramos, T; Lee D; Kim, Y.; Michaelides, A.; Oh, T.J.; Kim, K.M; Jang H.C.; Lim, S.;	Effectiveness of a Smartphone Application for the Management of Metabolic Syndrome Components Focusing on Weight Loss: A Preliminary Study	2017	1.597	Matched control	104	Weight; BP; Waist circumference; Glucose profile; Lipid profile; Body composition	No, no comparison to other treatment	-
Noom	Toro-Ramos, T.; Michaelides, A.; Anton, M.; Karim, Z.; Kang-Oh, L.; Argyrou, C.; Loukaidou, E.; Charitou, M.M.; Sze, W.; Miller, J.D.	Mobile Delivery of the Diabetes Prevention Program in People With Prediabetes: Randomized Controlled Trial	2020	4.301	RCT	202	HbA1c*; Weight*; BMI	Yes, compared to regular medical care	No difference in HbA1c levels between groups at 6 months (mean difference 0.004%, SE 0.05; P=.94) or 12 months (0.006%; SE 0.07; P=.93). At one year, the intervention group showed a 0.23% reduction in HbA1c levels while the control group had a 0.16% reduction in HbA1c. Weight and BMI in the intervention group were significantly lower at 6 months (mean difference -2.64kg, SE 0.71, P<0.001 and -0.99 kg/m^2, SE 0.29, P<0.001, respectively) and at 12 months (mean difference -1.80kg, SE 0.81, P=0.01 and -0.58kg/m^2, SE 0.24, P=0.01, respectively).
Noom	DeLuca, L.; Toro-Ramos, T.; Michaelides, A.; Seng, E.; Swencionis, C.	Relationship Between Age and Weight Loss in Noom: Quasi-Experimental Study	2020	4.532	NCT	14767	Weight	No, no comparison to other treatment	-

Noom	Chin, S.O.; Keum, C.; Woo, .J.; Park, J.; Choi, H.J.; Woo, J.; Rhee, S.Y.	Successful weight reduction and maintenance by using a smartphone application in those with overweight and obesity	2016	4.011	NCT	35921	Weight	No, no comparison to other treatment	-
Noom	Michaelides, A.; Major, J.; Pienkosz, E.; Wood, M.; Kim, Y.; Toro-Ramos, T.	Usefulness of a Novel Mobile Diabetes Prevention Program Delivery Platform With Human Coaching: 65-Week Observational Follow-Up	2018	4.301	NCT	140	Weight	No, no comparison to other treatment	-
Noom	Michaelides, A.; Raby, C.; Wood, M.; Farr, K.; Toro-Ramos, T.	Weight loss efficacy of a novel mobile Diabetes Prevention Program delivery platform with human coaching	2016	5.067	NCT	43	Weight	No, no comparison to other treatment	-
Sweetch	Everett, E.; Kane, B.; Yoo, A.; Dobs, A.; Mathioudakis, N.	A Novel Approach for Fully Automated, Personalized HealthCoaching for Adults with Prediabetes Pilot Clinical Trial	2018	4.945	NCT	55	Physical activity; Weight; BMI; HbA1c	No, no comparison to other treatment	-
Welldoc	Quinn, C.C.; Shardell, M.D.; Terrin, M.L.; Barr, E.A.; Ballew, S.H.; Gruber-Baldini, A.L.	Cluster-Randomized Trial of a Mobile Phone Personalized Behavioral Intervention for Blood Glucose Control	2011	15.27	RCT	163	HbA1c*; Diabetes Symptoms; Diabetes Distress; Depression; BP; Lipids	Yes, compared to usual care	Mean HbA1c declined by 1.9% in the the maximal treatment group and 0.7% in the usual care group, which results in a difference of 1.2% (P<0.001) over 12 months.
Welldoc	Quinn, C.C.; Clough, S.S.; Minor, J.M.; Lender, D.; Okafor, M.C.; Gruber-Baldini, A.	WellDoc™ Mobile Diabetes Management Randomized Controlled Trial: Change in Clinical and Behavioral Outcomes and Patient and Physician Satisfaction	2008	4.48	RCT	30	HbA1c*; Medication use; Depression; Diet; Self-care; Control issues	No, no comparison to other treatment	#not adequately powered
Welldoc	Quinn, C.C.; Shardell, M.D.; Terrin, M.L.; Barr, E.A.; Park, D.; Shaikh, F.; Guralnik, J.; Gruber-Baldini, A.L.	Mobile Diabetes Intervention for Glycemic Control in 45-to 64-Year-Old Persons With Type 2 Diabetes	2016	1.638	Secondary analysis of RCT	118	HbA1c	Yes, compared to usual care	-
Welldoc	Quinn, C.C.; Sareh, P.L.; Shardell, M.L.; Terrin, M.L.; Barr, E.A.; Gruber-Baldini, A.L.	Mobile Diabetes Intervention for Glycemic Control: Impact on Physician Prescribing	2014	NA	Secondary analysis of RCT	117	Medication use	Yes, compared to usual care	-
Welldoc	Desveaux, L.; Shaw, J.; Saragosa, M.; Soobiah, C.; Marani, H.; Hansel, J.; Agarwal, P.; Onabajo, N.; Bhatia, R.S.; Jeffs, L.	A Mobile App to Improve Self-Management of Individuals With Type 2 Diabetes: Qualitative Realist Evaluation	2018	5.03	NCT	16	HbA1c	No, no comparison to other treatment	-

Welldoc	Quinn, C.C.; Swasey, K.K.; Crabbe, C.F.; Shardell, M.D.; Terrin, M.L.; Barr, E.A.; Gruber-Baldini, A.L.	The Impact of a Mobile Diabetes Health Intervention on Diabetes Distress and Depression Among Adults: Secondary Analysis of a Cluster Randomized Controlled Trial	2017	4.313	Secondary analysis of RCT	163	Diabetes Distress; Depression	Yes, compared to usual care	-
Virta Health	Hallberg, S.J.; McKenzie, A.L.; Williams, P.T.; Bhanpuri, N.H.; Peters, A.L.; Campell, W.W.; Hazburn, T.L.; Volk, B.M.; McCarter, J.P.; Phinney, S.D.; Volek, J.S	Effectiveness and Safety of a Novel Care Model for the Management of Type 2 Diabetes at 1YearAn Open-Label, Non-Randomized, Controlled Study	2018	2.827	CT	349	HbA1c; Weight; Medication use; Blood glucose; HOMA-IR; Blood Lipids and lipoproteins; Liver and kidney function markers; C-reactive protein	Yes, compared to usual care	-
Virta Health	McKenzie, A.L.; Hallberg, S.J.; Creighton, B.C.; Volk, B.M.; Link, T.M.; Abner, M.K.; Glon, R.M.; McCartner, J.P.; Volek, J.S.; Phinney, S.D.	A Novel Intervention Including Individualized Nutritional Recommendations Reduces Hemoglobin A1c Level, Medication Use, and Weight in Type 2 Diabetes	2017	4.534	NCT	262	HbA1c; Weight; Medication use; BP; Cholesterol; Triglycerides; C-reactive protein; White blood cell count; Kidney and liver functions	No, no comparison to other treatment	-
Virta Health	Bhanpuri, N.H; Hallberg, S.J.; Williams, P.T.; McKenzie, A.L.; Ballard, K.D.; Campell, W.W.; McCarter, J.P.; Phinney, S.D.; Volek, J.S.;	Cardiovascular disease risk factor responses to a type 2 diabetes care model including nutritional ketosis induced by sustained carbohydrate restriction at 1 year: an open label, non-randomized, controlled study	2018	8.285	CT	262	Biomarkers of cholesterol metabolism and inflammation; BP; Carotid intima media thickness; Multi-factorial risk scores; Medication use	Yes, compared to usual care	-
Virta Health	Siegmann, M.J.; Athinarayanan, S.J.; Hallberg, S.J.; McKenzie, A.L.; Bhanpuri, N.H.; Campbell, W.W; McCarter, J.P.; Phinney, S.D.; Volek, J.S.; Van Dort, C.J	Improvement in patient-reported sleep in type 2 diabetes and prediabetes participants receiving a continuous care intervention withnutritional ketosis	2018	3.36	CT	262	Sleep quality	Yes, compared to usual care	-

Virta Health	Athinarayanan,S.J.; Adams, R.N.; Hallberg, S.J.; McKenzie, A.L.; Bhanpuri, N.H.; Campbell, W.W.; Volek, J.S.; Phinney, S.D; McCarter, J.P	Long-Term Effects of a Novel Continuous Remote Care Intervention Including Nutritional Ketosis for the Management of Type 2 Diabetes: A 2-Year Non-randomized Clinical Trial	2019	3.634	CT	262	HbA1c; HOMA-IR; Blood glucose; Fasting insulin; C-Peptide; Weight; Body composition; Liver, cardiovascular, thyroid, and inflammatory markers; Medication use; Disease status	Yes, compared to usual care	-
Virta Health	Athinarayanan, S.J.; Hallberg, S.J.; McKenzie, A.L.; Lechner, K.; King, S.; McCarter, J.P.; Volek, J.S.; Phinney, S.D.; Krauss, R.M.	Impact of a 2-year trial of nutritional ketosis on indices of cardiovascular disease risk in patients with type 2 diabetes	2020	7.332	CT	262	Risk markers of atherosclerotic cardiovascular disease	Yes, compared to usual care	-
Virta Health	McKenzie, A.L.; Athinarayanan,S.J.; McCue, J.J.; Adams, R.N.; Keyes, M.; McCarter, J.P.; Volek, J.S.; Phinney, S.D.; Hallberg, S.J.	Type 2 Diabetes Prevention Focused on Normalization of Glycemia: A Two-Year Pilot Study	2021	4.546	NCT	96	Change in metabolic status	No, no comparison to other treatment	-
Oviva	Haas, K.; Hayot, S.; Maurer-Wiesner, S.	Effectiveness and Feasibility of a Remote Lifestyle Intervention by Dietitians for Overweight and Obese Adults: Pilot Study	2019	4.31	NCT	43	Weight; HbA1c; Blood glucose; Fasting insulin, triglyceride, HDL cholesterol; BP; BMI; waist circumference; Body fat; Physical activity; Diet; Quality of life	No, no comparison to other treatment	-
Oviva	Huntriss, R.; Haines, M.; Jones, L.; Mulligan, D.	A service evaluation exploring the effectiveness of a locally commissioned tier 3 weight management programme offering face-to-face, telephone and digital dietetic support	2021	NA	NCT	169	Weight; BMI; Number of psychology support sessions	Yes, compared to face-to-face support and telephone support	-
Livongo	Bollyky, J.B.; Bravata, D.; Yang, J.; Williamson, M.; Schneider, J.	Remote Lifestyle Coaching Plus a Connected Glucose Meter with Certified Diabetes Educator Support Improves Glucose and Weight Loss for People with Type 2 Diabetes	2018	3.04	CT	330	Blood glucose; HbA1c; Weight	Yes, compared to light coaching and to receiving only a connected scale	-

Livongo	Bollyky, J.B.; Melton, S.T.; Xu, T.; Painter, S.L.; Knox, B.	The Effect of a Cellular-Enabled Glucose Meter on Glucose Control for Patients With Diabetes: Prospective Pre-Post Study	2019	4.532	NCT	86	HbA1c; Blood glucose; Depression	No, no comparison to other treatment	-
Livongo	Downing, J.; Bollyky, J.; Schneider, J.	Use of a Connected Glucose Meter and Certified Diabetes Educator Coaching to Decrease the Likelihood of Abnormal Blood Glucose Excursions: The Livongo for Diabetes Program	2017	4.945	NCT	4544	Blood glucose	No, no comparison to other treatment	-
Livongo	Amante, D.J.; Harlan, D.M.; Lemon, S.C.; McManus, D.D.; Olaitan, O.O.; Pagoto, S.L.; Gerber, B.S.; Thompson, M.J.	Evaluation of a Diabetes Remote Monitoring Program Facilitated by Connected Glucose Meters for Patients With Poorly Controlled Type 2 Diabetes: Randomized Crossover Trial	2021	4.532	RCT	119	HbA1c*	Yes, comparison to usual care	- Improvements in mean HbA1c seen in both groups during the first 6 months (Intervention (IV)/Usual Care (US) -1.1% , SD 1.5 vs UC/IV -0.8%, SD 1.5; P<.001). After cross-over, no change in HbA1c of IV/UC (mean HbA1c change +0.2, SD 1.7, P=.41), but further improvement in UC/IV (mean HbA1c change -0.4%, SD 1.0, P=.008).<br>- Mixed-effects model revealed a nonsignificant difference in HbA1c improvement of 0.37% between the intervention and ususal care treatment groups over 12 months
Liva Health	Komkova, A.; Brandt, C.J.; Pedersen, D.H.; Emneus, M.; Sortsø, C.	Electronic Health Lifestyle Coaching Among Diabetes Patients in a Real-Life Municipality Setting: Observational Study	2019	4.532	NCT	103	Weight	No, no comparison to other treatment	-
Liva Health	Brandt, V.; Brandt, C.J.; Glingtborg, D.; Arendal, C.; Tourbo, S.; Brandt, K.	Sustained Weight Loss during 20 Months using a Personalized Interactive Internet Based Dietician Advice Program in a General Practice Setting	2011	0.14	NCT	32	Weight	No, no comparison to other treatment	-
Liva Health	Haste, A.; Adamson, A.J.; McColl, E.; Araujo-Soares, V.; Bell, R.	Web-Based Weight Loss Intervention for Men With Type 2 Diabetes: Pilot Randomized Controlled Trial	2017	4.532	RCT	61	Weight; BMI; Waist circumference	No, no comparison to other treatment	#not adequately powered

Liva Health	Johansen, M.Y.; MacDonald, C.S.; Hansen, K.B.; Karstoft, K.; Christensen, R.; Pedersen, M.; Hansen, L.S.; Zacho, M.; Wedell-Neergaard A.; Nielsen, S.T.; Iepsen, U.W.; Langberg, H.; Vaag, A.A.; Pedersen, B.K.; Ried-Larsen, M.	Effect of an Intensive Lifestyle Intervention on Glycemic Control in Patients With Type 2 Diabetes A Randomized Clinical Trial	2017	45.54	RCT	98	HbA1c*; Medication use; Lipids, BP; Body composition, Physical fitness, Physical activity, Diet	No, no comparison to other treatment	Over 12 months, the mean HbA1c level decreased by 0.31% in the lifestyle group, compared 0.04% in the standard care group. The between-group difference did not meet the predefined equivalence of ±0.4% for a clinically relevant reduction in HbA1c level (mean between-group difference of -0.26% [95% CI, -0.52% to -0.01], P=0.15).
Lark	Stein, N.; Brooks, K.	A Fully Automated Conversational Artificial Intelligence for Weight Loss: Longitudinal Observational Study Among Overweight and Obese Adults	2017	4.532	NCT	70	Weight; Meal quality	No, no comparison to other treatment	-
Holmusk	Koot, D.; Goh, P.S.C.; Lim, R.S.M.; Tian, Y.; Yau, T.Y.; Tan, N.C.; Finkelstein, E.A.	A Mobile Lifestyle Management Program (GlycoLeap) for People With Type 2 Diabetes: Single-Arm Feasibility Study	2019	4.313	NCT	100	HbA1c; Weight	No, no comparison to other treatment	-
Vida Health	Mao, A.Y.; Chen, C.; Magana, C.; Barajas, K.C.; Olayiwola, N.	A Mobile Phone-Based Health Coaching Intervention for Weight Loss and Blood Pressure Reduction in a National Payer Population: A Retrospective Study	2017	4.301	Matched pair-control	1012	Weight; BP	No, compared to standard pregnancy dietary orientation related to gestational weight gain but not diabetes	-
Vida Health	Silberman, J.M.; Kaur, M.; Sletteland, J.; Venkatesan, A.	Outcomes in a digital weight management intervention with one-on-one health coaching	2019	2.776	NCT	683	Weight	No, no comparison to other treatment	-
One Drop	Osborn, C.Y.; Van Ginkel, J.R.; Marrero, D.G.; Rodbard, D.; Huddleston, B.; Dachis, J.	One Drop Mobile on iPhone and Apple Watch An Evaluation of HbA1c Improvement Associated With Tracking Self-Care	2017	4.301	NCT	256	HbA1c	No, no comparison to other treatment	-
One Drop	Osborn, C.Y.; Van Ginkel, J.R.; Rodbard, D.; Heyman, M.; Marrero, D.; Huddleston, B.; Dachis, J.	One Drop | Mobile: An Evaluation of Hemoglobin A1c Improvement Linked to App Engagement	2017	4.532	NCT	1288	HbA1c	No, no comparison to other treatment	-

Twin Health	Shamanna, P.; Saboo, B.; Damodharan, S.; Mohammed, J.; Mohamed, M.; Poon, T.; Kleinman, N.; Thajudeen, M.	Reducing HbA1c in Type 2 Diabetes Using Digital Twin Technology-Enabled Precision Nutrition: A Retrospective Analysis	2020	3.179	NCT	64	HbA1c; Blood glucose; HOMA-IR; C-Peptide, Sleep, Physical activity; Heart rate	No, no comparison to other treatment	-
Omada Health	Chen, F.; Su, W.; Becker, S.H.; Payne, M.; Sweet, C.M.C.; Peters, A.L.; Dall, T.M.	Clinical and Economic Impact of a Digital, Remotely-Delivered Intensive Behavioral Counseling Program on Medicare Beneficiaries at Risk for Diabetes and Cardiovascular Disease	2016	2.776	NCT	1121	Weight	No, no comparison to other treatment	-
Omada Health	Sepah, S.C.; Jiang, L.; Ellis, R.J.; McDermott, K.; Peters, A.L.	Engagement and outcomes in a digital Diabetes Prevention Program: 3-year update	2017	3.183	NCT	220	Weight; HbA1c	No, no comparison to other treatment	-
Omada Health	Wilson, ,.G.; Sweet, C.M.C.; Edge, M.D.; Madero, E.N.; McGuire, M.; Pilsmaker, M.; Carpenter, D.; Kirschner, S.	Evaluation of a Digital Behavioral Counseling Program for Reducing Risk Factors for Chronic Disease in a Workforce	2017	1.591	Matched control	1902	Weight; BMI; Blood glucose; BP; Lipids; Waist circumference; Health behaviours	No, no comparison to other treatment	-
Omada Health	Sepah, S.C.; Jiang, L.; Peters, A.L.	Long-Term Outcomes of a Web-Based Diabetes Prevention Program: 2-Year Results of a Single-Arm Longitudinal Study	2015	4.945	NCT	254	Weight; HbA1c	No, no comparison to other treatment	-
Omada Health	Sweet, C.C.; Chiguluri, V.; Gumpina, R.; Abbott, P.; Madero, E.N.; Payne, M.; Happe, L.; Matanich, R.; Renda, A.; Prewitt, T.	Outcomes of a Digital Health Program With Human Coaching for Diabetes Risk Reduction in a Medicare Population	2017	2.007	NCT	9498	Weight; HbA1c; Cholesterol; Depression; Well-being	No, no comparison to other treatment	-
Omada Health	Moin, T.; Damschroder, L.; AuYong, M.; Maciejewski, M.L.; Havens, K.; Ertl, K.; Vasti, E.; Weinreb, J.E.; Steinle, N.; Billington, C.J.; Hughes, M.; Makki, F.; Youles, B.; Holleman, R.G.; Kim, H.M.; Kinsinger, L.S.; Richardson, C.R.	Results From a Trial of an Online Diabetes Prevention Program Intervention	2018	4.435	CT	268	Weight	Yes, results compared to one in-person diabetes prevention program and to one weight loss program from a separate trial	-
Omada Health	Su, W.; Chen, F.; Dall, T.M.; Iacobucci, W.; Perreault, L.	Return on Investment for Digital Behavioral Counseling in Patients With Prediabetes and Cardiovascular Disease	2016	2.038	Matched control	1663	Weight; Diabetes incidence	No, no comparison to other treatment	-

Omada Health	Sepah, S.C.; Jiang, L.; Peters, A.L.	Translating the Diabetes Prevention Program into an Online Social Network: Validation against CDC Standards	2014	2.087	NCT	220	Weight; HbA1c	No, no comparison to other treatment	-
Omada Health	Moin, T.; Ertl, K.; Schneider, J.; Vasti, E.; Makki, F.; Richardson, C.; Havens, K.; Damschroder, L.	Women Veterans' Experience With a Web-Based Diabetes Prevention Program: A Qualitative Study to Inform Future Practice	2015	4.945	NCT	20	Weight	No, no comparison to other treatment	-
Omada Health	Sweet, C.C; Jasik, C.B.; Diebold, A.; DuPuis, A.; Jenretzke, B.	Cost Savings and Reduced Health Care Utilization Associated with Participation in a Digital Diabetes Prevention Program in an Adult Workforce Population	2020	NA	Matched control	2027	Weight	No, no comparison to other treatment	-
Omada Health	Wilson-Anumudu, F.; Quan, R.; Sweet, C.C.; Cerrada, C.; Juusola, J.; Turken, M.; Jasik, C.B.	Early Insights From a Digitally Enhanced Diabetes Self-Management Education and Support Program: Single-Arm Nonrandomized Trial	2021	4.532	NCT	195	HbA1c; Lipids; Diabetes distress; Medication use	No, no comparison to other treatment	-

Abbreviations: DBCI=Digital behaviour change intervention*=Primary outcomes (only in RCTs); #=RCT not adequately powered; NA=Not available; BMI=body mass index; BP=Blood pressure; CT=Controlled trial; NCT=Non-controlled trial; RCT=Randomized-controlled trial
